# Changes in sleep quality during the 12 months following medical cannabis initiation

**DOI:** 10.1186/s42238-025-00376-7

**Published:** 2025-12-20

**Authors:** Megan M. Short, Michelle R. Lent, Thomas R. McCalmont, Karen L. Dugosh

**Affiliations:** 1 Department of Clinical Psychology, Philadelphia College of Osteopathic Medicine, 4190 City Avenue, Philadelphia, PA 19131 USA; 2 Research & Evaluation Group, Public Health Management Corporation, Philadelphia, PA USA

**Keywords:** Medical cannabis, Sleep quality, Medical marijuana

## Abstract

**Background:**

Poor sleep quality is a commonly reported reason for medical cannabis (MC) use, yet evidence regarding its long-term impact on sleep remains limited. This study evaluated changes in subjective sleep quality over a 12-month period among adults initiating MC treatment in Pennsylvania and explored whether preferred route of administration and referring condition were associated with observed changes.

**Methods:**

A total of 137 adults newly referred for MC in PA completed the Pittsburgh Sleep Quality Index (PSQI) at baseline and at 3, 6, 9, and 12 months. Linear mixed effects models assessed changes in PSQI global and subscale scores over time. Additional models evaluated whether preferred administration route (oral vs. other) and referring condition (chronic pain, anxiety, PTSD) were associated with differences in observed outcomes.

**Results:**

Global sleep quality scores, where higher values indicate poorer sleep quality, were significantly higher at baseline than at each follow-up point (*p* < .0001), with no significant differences among follow-up assessments, suggesting early and sustained improvements in self-reported sleep quality. Improvements were observed across all PSQI subscales. No significant relationships were found between sleep quality scores and either administration route or referring condition.

**Conclusions:**

These findings suggest that MC may be associated with improvements in subjective sleep quality, though its impact did not vary as a function of administration route or primary referring condition. Additional research using objective sleep measures and controlled designs is needed to clarify MC’s role in sleep quality.

The use of medical cannabis (MC) has increased steadily in recent years, largely due to expanding state-level legalization and increased awareness of its potential in treating conditions like chronic pain, anxiety, and posttraumatic stress disorder (PTSD) (Boehnke et al. 2022; Rhee and Rosenheck 2023). MC patients who have these conditions frequently cite poor sleep quality, characterized by difficulty initiating or maintaining sleep, as a primary symptom they hope to address with MC use (Babson et al. 2017; Kimless et al. 2022). Despite widespread use, empirical evidence evaluating the relationship between MC use and sleep quality, particularly over extended periods of use, remains limited and existing findings are mixed.

Several observational studies have reported improvements in subjective sleep quality within the first six months following MC initiation, including reductions in sleep latency and longer total sleep duration, particularly among individuals with chronic pain or PTSD (Ergisi et al. 2022; Vaddiparti et al. 2023). For example, a retrospective study by Tringale & Jenson (Tringale and Jensen 2011) found that MC patients, on average, fell asleep approximately thirty minutes faster than they had prior to initiating MC use, even if they did not report sleep issues at baseline. However, other observational findings have associated frequency of cannabis use with poorer overall sleep quality (Ogeil et al. 2015; Winiger et al. 2021). These findings suggest that while some individuals may experience initial improvements in sleep after starting MC, benefits may not be consistent nor sustained. While data from randomized controlled trials are limited given the Schedule I designation of cannabis in the United States, Ware et al. (Ware et al. 2010) found that nabilone, a synthetic cannabinoid that mimics tetrahydrocannabinol (THC), improved sleep more effectively than amitriptyline among patients with fibromyalgia. Nevertheless, concerns remain about potential adverse effects of cannabis use, including daytime drowsiness and other dose-related side effects (Babson et al. 2017; Suraev et al. 2020). Few studies to date have systematically evaluated long-term changes in sleep quality following MC initiation.

The impact of long-term cannabis use on sleep may vary depending on the context of use. Chronic recreational or problematic use has been associated with poorer sleep quality and increased sleep disturbances, whereas targeted medicinal use has been linked to short-term improvements in sleep latency and duration (Babson et al. 2017; O’Brien et al. 2021; Poyatos et al. 2020). Pharmacologic factors may also play a role. Cannabidiol (CBD) exhibits lower oral bioavailability than tetrahydrocannabinol (THC), which may partly explain variability in treatment response (O’Brien et al. 2021; Ogeil et al. 2015). Additionally, emerging research suggests that combined CBD and THC formulations may produce differential outcomes depending on cannabinoid ratios, dosing, and route of administration (O’Brien et al. 2021).

Route of administration is another important consideration impacting the, onset, duration, and intensity of MC effects (O’Brien et al. 2021; Poyatos et al. 2020), which may have important implications for sleep. Oral formulations of MC, including capsules, oils, tinctures, and troches, are associated with slower absorption and delayed onset but longer duration compared with inhaled routes (Poyatos et al. 2020). This extended duration of action may be particularly beneficial for individuals using MC to improve sleep quality, as it may support sustained symptom relief throughout the night. In fact, a randomized, placebo-controlled crossover trial by Walsh et al. (Walsh et al. 2021) found that nightly administration of an oral sublingual cannabinoid formulation containing THC, cannabidiol (CBD), and cannabinol (CBN) was associated with significant improvements in self-reported sleep onset and total sleep time among individuals with chronic insomnia. These findings suggest that oral MC formulations may be especially useful for sustaining sleep quality. Patients who express a preference for oral MC products may be selecting these formulations to achieve longer-lasting effects; thus, route preference may be an important factor to consider when evaluating sleep quality among MC patients.

This longitudinal, observational study evaluated changes in subjective sleep quality over a 12-month period among adults initiating MC treatment for one or more of the 24 qualifying medical conditions in Pennsylvania. At the time of participant recruitment, Pennsylvania permitted MC use but not recreational use (2016 Act 16 n.d.). The Pennsylvania Medical Marijuana Act (Act 16) authorizes access to MC for individuals certified by a Pennsylvania Department of Health-approved physician for one or more of 24 qualifying medical conditions (Department of Health, Commonwealth of Pennsylvania 2025; 2016 Act 16 n.d.). Certified patients receive a state-issued identification card (“medical marijuana card”) and may purchase MC products only from licensed dispensaries (Act 16 n.d.). Each dispensary is required to have a pharmacist or physician available on-site to verify patient certifications and provide product guidance (2016 Act 16 n.d.).

Global sleep quality scores were collected at baseline and at three-month follow-up intervals for one year post-baseline. We hypothesized that (1) global sleep quality scores would improve over the 12-month period following MC initiation; and (2) individuals preferring orally administered MC products (i.e., capsules, extracts, tinctures, and troches)would endorse greater improvements in global sleep quality scores compared to those who preferred other routes.

## Methods

### Sample

Participants included 137 adults residing in Pennsylvania, aged 18 years and older who were newly referred (i.e., within the two weeks prior to enrollment) for MC treatment for a variety of health conditions and participating in a larger study evaluating the relationship between MC initiation and biopsychosocial functioning over the first year of MC use (Lent et al. 2024). To be eligible for participation in the larger study, participants must have been (1) able to read and understand English, (2) able to provide informed consent, (3) holder of a valid PA MC card, and (4) willing to participate in follow-up assessments over the next 12 months. To be eligible for the current analyses, individuals had to (1) report sleep disorders as a secondary condition they hoped to treat with MC, (2) report a global sleep quality score of greater than 5 as measured by the Pittsburgh Sleep Quality Index (PSQI) (Buysse et al. 1989) and (3) complete at least one follow-up assessment. Of 462 individuals enrolled in the larger study, 137 participants met inclusion criteria and were included for the current analyses. All participants included in this analytic sample reported being active medical cannabis users at each time point during the 12-month study period.

### Measures

#### Demographic and MC information

Participants reported their biological sex, age, marital status, race/ethnicity, and education level in a demographic questionnaire developed by study investigators. Additionally, participants reported their medical reason for their MC recommendation and secondary health concerns they hoped to treat with MC. At follow-up assessments, after MC initiation, participants reported their preferred route of administration for MC products.

#### Pittsburgh Sleep Quality Index (PSQI)

Sleep quality was assessed using the Pittsburgh Sleep Quality Index (PSQI) (Buysse et al. 1989), a well-validated self-report questionnaire designed to measure sleep quality over a one-month interval. The PSQI consists of 19 self-rated items that produce seven component scores: subjective sleep quality, sleep latency, sleep duration, habitual sleep efficiency, sleep disturbances, use of sleeping medications, and daytime dysfunction. Each component is scored on a 0 to 3 scale, with higher scores indicating more significant sleep disturbances. Component scores are summed to produce a global score ranging from 0 to 21. A global score greater than 5 is typically indicative of poor sleep quality.

### Procedure

Participants were recruited from four medical cannabis dispensaries (Organic Remedies, Inc.) located in Western and Central Pennsylvania as part of a larger longitudinal study examining MC use and quality of life (Lent et al. 2024). After providing informed consent, eligible participants completed a baseline assessment that included demographic information, questions about participants’ referring condition(s) for MC, and the PSQI. Participants then initiated MC treatment. Follow-up assessments were administered by research staff at 3, 6, 9, and 12 months post-baseline. At each time point, participants completed the PSQI and a brief questionnaire capturing information on preferred MC product type and preferred route of administration (e.g., inhaled, oral, tincture). Study data were collected and managed using REDCAP (Research Electronic Data Capture) hosted at the lead institution. REDCap is a secure, web-based software platform designed to support data capture for research studies (Harris et al. 2009). Participants received a $25 debit card payment for completing each study visit. This study was not preregistered. This study was conducted with the approval and ethical oversight of the Philadelphia College of Osteopathic Medicine’s Institutional Review Board (#H17–060) and was conducted in accordance with the ethical principles outlined in the Declaration of Helsinki.

### Data analysis

Descriptive statistics were used to characterize the sample at baseline. For the primary hypothesis, a series of linear mixed effects models were used to examine changes over time in PSQI global score and each of its 7 sub-scale scores. Each model included term for time (0, 3, 6, 9, 12) and accounted for the nesting of responses within individual. Mixed effects models have advantages over conventional repeated-measures methods in that they allow for missing observations, accommodate longitudinal measurement, and provide greater flexibility in modeling the variance–covariance matrix (Diggle et al. 2002). For the secondary hypotheses related to preference, a linear mixed effects model was used to examine group differences over time in PSQI global score after controlling for baseline score. The model included a binary term for oral preference (i.e., oral vs. other preferred route of administration), time (3, 6, 9, 12), and the oral preference by time interaction, and baseline score as a covariate. Products administered orally include capsules, extracts, tinctures, and troches. Because the preferred route of administration was assessed at each follow-up point, participants could indicate a different preference at each timepoint. Oral preference in the reported analysis was defined as indicating preference for orally administered products at 50% or more of the completed follow-up assessments. Other approaches (i.e., reported oral preference at one or more follow-up assessment and oral preference as a time varying variable) resulted in similar results to those reported below and, for this reason, are not reported in the manuscript. For the secondary hypothesis related to primary referring condition, a series of mixed effects models were used to examine changes over time as a function of referring condition. Separate models generated for each of the three most reported referring conditions in the sample [i.e., chronic pain, anxiety, and post-traumatic stress disorder (PTSD)], all conditions characterized by significant sleep impairment. Each model included a binary term for the specific diagnosis (present vs. absent), time, the diagnosis by time interaction, and the baseline score as a covariate. All models described above specified a compound symmetry covariance structure. All analyses were performed using SAS® 9.4 statistical software.

## Results

### Participants

Demographic and baseline status information for the analytic sample is presented in Table 1. The majority of participants identified as female (*n* = 85, 62%), White (*n* = 126, 92%), and non-Hispanic or Latinx (*n* = 132, 96%) with an average age of 43.48 years (*SD* = 15.91). The most frequently reported referring conditions were anxiety disorders (*n* = 94, 69%), severe chronic or intractable pain (*n* = 59, 43%) and PTSD (*n* = 27, 19%). The majority of participants (*n* = 78, 61%) reported no recreational cannabis use in the 90 days prior to study enrollment. Follow up completion rates at months 3, 6, 9, and 12 were 80% (n = 110), 69% (*n* = 95), 64% (*n* = 87), and 55% (*n* = 76), respectively.

**Table 1:** Demographic and clinical characteristics of the analytic sample (*n* = 137)

*Characteristic*	*Mean (SD)*	*N (%)*
Age (years)	43.48 (15.91)	
Sex assigned at birth		
Female		89 (64.96%)
Male		48 (35.04%)
Race		
White		126 (91.97%)
Black or African American		6 (4.38%)
Asian		3 (2.19%)
American Indian or Alaska Native		1 (0.73%)
Native Hawaiian or Pacific Islander		1 (0.73%)
Mixed race		4 (2.92%)
Ethnicity		
Hispanic or Latinx		5 (3.65%)
Not Hispanic or Latinx		132 (96.35%)
Education level		
High school diploma or less		28 (20.44%)
Some college, associate, or trade degree		55 (40.15%
Bachelor’s degree		34 (24.82%)
Graduate degree (Master’s or Doctorate)		20 (14.60%)
Marital status		
Single (never married)		43 (31.39%)
Married		62 (45.26%)
Other (partnered, separated, divorced, widowed)		32 (23.36%)
Recreational cannabis use (past 90 days)		
No use reported		78 (60.94%)
Reported use		50 (39.06%)
Lifetime recreational cannabis use (years)	11.46 (9.51)	
Primary referring conditions		
Anxiety disorders		94 (68.61%)
Chronic pain		59 (43.07%)
Posttraumatic stress disorder (PTSD)		27 (19.71%)

Some participants endorsed more than one referring condition

### PSQI global score

Results from the linear mixed effects model (*n* = *137)* indicated a significant effect of time, *F*(4, 359) = 38.65, *p* < 0.0001. Pairwise comparisons of least square means indicated that PSQI global scores were significantly higher at baseline than at months 3, 6, 9, and 12. Scores at the four follow-up assessments were not significantly different from one another. Mean scores at each timepoint are presented in Table 2.

**Table 2:** Models for the primary hypothesis (*n* = 137)

	Month					Time effect for model		
	0	3	6	9	12			
PSQI scale score	*M* *(SD)*	*M* *(SD)*	*M* *(SD)*	*M* *(SD*)	*M* *(SD)*	df	*F*	*p*
Global	10.89 (3.84)^a^	7.61 (3.66)^b^	7.27 (3.55)^b^	7.83 (4.21)^b^	7.53 (4.07)^b^	4, 359	38.65	< .0001
Duration	1.47 (1.10)^a^	0.90 (0.99)^b^	0.87 (1.03)^b^	1.05 (1.08)^b^	1.01 (1.10)^b^	4, 364	14.88	< .0001
Disturbance	1.66 (0.56)^a^	1.38 (0.49)^b^	1.34 (0.63)^b^	1.37 (0.60)	1.37 (0.59)^b^	4, 360	10.34	< .0001
Latency	2.01 (1.07)^a^	1.47 (1.14)^b^	1.51 (1.09)^b^	1.45 (1.10)	1.33 (1.17)^b^	4, 364	15.41	< .0001
Daytime disfunction	1.11 (0.76)^a^	0.75 (0.67)^b^	0.64 (0.70)^b^	0.72 (0.68)	0.59 (0.66)^b^	4, 364	14.62	< .0001
Efficiency	1.56 (1.17)^a^	1.05 (1.10)^b^	1.01 (1.07)^b^	1.21 (1.21)	1.17 (1.17)^b^	4, 364	5.71	< .001
Overall quality	1.79 (0.80)^a^	1.22 (0.76)^b^	1.26 (0.76)^b^	0.78 (0.85)	1.17 (0.77)^b^	4, 363	22.87	< .0001
Medication	1.30 (1.34)^a^	0.86 (1.22)^b^	0.63 (1.11)^c^	0.78 (1.20)^bc^	0.84 (1.24)^bc^	4, 364	11.21	< .0001

For each outcome measure, means with different letters are significantly different from one another

### PSQI subscale scores

As seen in Table 2, similar results were observed for each of the PSQI subscales with significantly higher scores observed at baseline than at any of the follow-up points across all domains. For the model predicting PSQI sleep medication score, pairwise comparisons additionally indicated that month 3 score was significantly higher than month 6 score, indicating further improvement from month 3 to 6 for this domain.

### PSQI global scores as a function of preferred route of administration (Oral vs. Other)

Of those participants who completed at least one follow-up assessment and, consequently, provided information about their preferred route of administration (*n* = 112), 41% (*n* = 46) reported preferring orally administered products. Results from the linear mixed effects model comparing PSQI global scores between those who did and did not have an oral preference (*n* = 110) revealed only a significant effect of time, *F*(4, 354) = 32.45, *p* < 0.0001. Oral preference and the oral preference by time interaction effects were not statistically significant in the model, *p*’s = 0.73 and 0.93, respectively. Group means at each timepoint are presented in Table 2.

### PSQI global scores as a function of referring condition

Results of the series of linear mixed effects models comparing PSQI global scores as a function of referring condition (i.e., anxiety, chronic pain, and PTSD) indicated that changes in overall sleep quality from baseline to follow-up did not vary as a function of condition as there was no condition by time interaction in any of the models (*p*’s = 0.21, 0.75, and 0.37, respectively). In the anxiety and chronic pain models, time was the only significant effect in the model, *F*(4, 355) = 29.02, *p* < 0.0001 and *F*(4, 355) = 35.85, *p* < 0.0001, respectively, with significantly higher scores at baseline than at the follow-up assessments in each model. For the PTSD model, both time, *F*(4, 355) = 27.93, *p* < 0.0001, and condition,* F*(1, 135) = 7.42, *p* < 0.01, reached statistical significance. Specifically, scores were significantly higher at baseline than at the follow-up assessments and scores for participants with PTSD as a referring condition had poorer overall sleep quality than their counterparts.

## Discussion

This prospective, longitudinal study evaluated changes in subjective sleep quality over 12 months among adults initiating MC for any of the 24 approved qualifying conditions in Pennsylvania (Department of Health, Commonwealth of Pennsylvania 2025). As hypothesized, results indicated significant improvements in global sleep quality scores following MC initiation, with the most substantial changes (i.e., a 30.1% decrease) observed between baseline and the three-month assessment. These improvements were sustained across the remainder of the study period, with no significant differences between follow-up intervals.

Our findings align with previous observational research indicating early improvements in subjective sleep quality following MC initiation. For example, Vaddiparti et al. (Rhee and Rosenheck 2023) reported significant reductions in sleep disturbances among individuals with PTSD within three months of MC use. Similarly, Ergisi et al. (2022) observed improvements in sleep latency and duration among MC users in the UK Medical Cannabis Registry. Our results extend these findings by indicating that self-reported improvements in sleep quality were sustained over longer time periods overall and across the specific domains measured by the PSQI.

Although we hypothesized that the route of administration would impact the relationship between MC use and sleep quality, this hypothesis was not supported. Participants who expressed a preference for orally administered products did not report greater improvements in sleep quality relative to those who preferred other administration methods. This result diverges from findings by Bidwell et al. (Bidwell et al. 2024), who suggested that oral cannabis products, particularly those with high CBD concentrations, may have longer-lasting sleep benefits. One possible explanation for our divergent finding is that MC users in our study may have frequently varied their product formulations and routes based on changing symptoms or product availability, making consistent categorization difficult. These variations may have included differences in product type or cannabinoid composition (e.g., THC- versus CBD-dominant formulations), though detailed composition data were not analyzed in the present study. Future research could benefit from capturing more detailed data oncomposition, dose, frequency of use, and administration methods to better understand how these factors impact sleep quality.

We also found no significant relationship between referring condition and change in sleep quality over time, although participants with PTSD reported significantly poorer sleep quality overall than their counterparts. These findings are partially consistent with prior work by Nacasch et al. (Nacasch et al. 2023) and Vaddiparti et al. (2023), who both observed significant improvements in sleep quality in individuals with PTSD using MC, though baseline sleep impairment was also notably high in those samples. Our findings suggest that although individuals with PTSD may benefit from MC, they continue to experience poorer sleep quality relative to individuals referred for other conditions, underscoring the need for integrated approaches to addressing sleep-related issues in this population.

The observed improvements in global sleep quality scores were consistent across all seven PSQI subdomains, including sleep latency, duration, and disturbances. This finding is notable given previous concerns regarding potential tolerance or diminished sleep benefits with long-term THC use (Babson et al. 2017; Suraev et al. 2020). It is possible that participants adjusted their dosing or product selection over time in response to changing symptoms or preferences; however, dose data were not collected in this study, and we cannot determine whether these changes impacted tolerance or perceived benefit. Future studies should include standardized assessments of cannabinoid content, dosing, and tolerance development to clarify these relationships.

There are several limitations of this study. First, the study relied exclusively on self-reported sleep quality, which may be influenced by expectancy and selection bias. Individuals seeking MC treatment for conditions such as pain, anxiety, or sleep disturbances may already believe that cannabis improves sleep, potentially inflating perceived benefits. Future studies should incorporate objective measures such as actigraphy or polysomnography to validate self-reported findings. Second, although all participants reported being active MC users at each follow-up point, detailed information on use frequency, timing of administration, dose, and cannabinoid ratios was not collected. These factors likely affect onset, duration, and overall sleep outcomes and should be evaluated in future research. Third, data on concurrent treatments (e.g., pharmacologic or behavioral therapies) for participants’ qualifying conditions as well as the duration of their qualifying condition(s) were not systematically collected. Improvements in these conditions may have indirectly contributed to perceived improvements in sleep quality. Fourth, participant attrition may have introduced bias, as individuals who discontinued MC use or experienced limited benefit may have been less likely to complete later assessments. Fifth, the sample was predominantly White and female, which may limit generalizability to more diverse populations. Finally, the observational design precludes causal inference; randomized controlled trials are needed to establish the efficacy of MC for improving sleep quality.

Despite these limitations, this study contributes to the growing body of literature exploring the potential role of MC in improving subjective sleep quality among individuals with chronic health conditions. Observed improvements across PSQI subdomains, including sleep latency, duration, and disturbances, may suggest clinical relevance, particularly for populations in which traditional treatments are insufficient or poorly tolerated. While these findings point to the therapeutic potential of MC for individuals experiencing poor sleep quality, continued research is needed to establish its effectiveness and inform clinical guidelines and best practices for MC use in sleep management.

## Conclusions

This observational, longitudinal study found that MC use was associated with sustained improvements in subjective sleep quality over a 12-month period. These improvements were consistent across all sleep domains and did not vary based on preferred route of administration or referring conditions. Findings contribute to the growing body of literature supporting the potential medicinal benefits of MC for sleep quality, particularly among individuals with chronic health conditions.

## Data Availability

Study data and a data dictionary are available at https://digitalcommons.pcom.edu/.

## Abbreviations

MC: Medical Cannabis
PTSD: Posttraumatic Stress Disorder
PSQI: Pittsburgh Sleep Quality Index
THC: Tetrahydrocannabinol
CBD: Cannabidiol
CBN: Cannabinol
REDCap: Research Electronic Data Capture
